# Inconclusive results of slow cortical potential neurofeedback for the treatment of chronic post-stroke attention deficits

**DOI:** 10.3389/fnhum.2024.1301622

**Published:** 2024-04-05

**Authors:** Sonja C. Kleih, Loic Botrel

**Affiliations:** Institute of Psychology, Biological Psychology Clinical Psychology and Psychotherapy, Faculty of Human Sciences, Julius-Maximilians-Universität Würzburg, Würzburg, Germany

**Keywords:** slow cortical potentials (SCP), neurofeedback, stroke, attention deficit, rehabilitation

## Abstract

**Introduction:**

Individuals who have suffered a stroke may experience long-lasting cognitive impairments that can worsen if left untreated. We investigated whether voluntary control of slow cortical potentials (SCP) through neurofeedback would help alleviate chronic post-stroke symptoms of impaired attention.

**Methods:**

The study initially enrolled twenty-eight participants, but due to a high drop-out rate, only sixteen participants completed eight SCP neurofeedback training sessions within three to four weeks. During these sessions, we gave feedback to the participants on their ability to regulate SCPs on a computer screen.

**Results:**

Our findings showed a non-significant increase in SCP regulation towards cortical negativity. On the behavioral level, we found improved test values in the divided attention and attentional flexibility subtests of the test battery for attention performance. However, we cannot eliminate the possibility that nonspecific effects influenced or caused our results. We have not observed any improvement regarding the effects of attention deficits on participants’ daily lives. We identified five individuals who could gain SCP control successfully and consistently towards negativity. In this group of responders, we observed an improvement in the test results related to divided attention but no other attention-related improvements.

**Discussion:**

Based on our observations, results of SCP neurofeedback training for the treatment of attention deficits after a stroke are inconclusive. More research is necessary to determine the effectiveness of SCP neurofeedback in helping stroke survivors cope with attention-related challenges in their daily lives.

## 1 Introduction

Increased health risks, such as stroke, accompany an aging society (Kolominsky-Rabas et al., 2006; Feigin et al., 2022). While in Europe in the year 2000 an absolute number of 1.1 million cases per year were reported, projections estimate 1.5 million cases already in 2025 with a rising trend (Béjot et al., 2016). Among the main risk factors are male sex, a sedentary lifestyle, hypertension, diabetes, a family history of stroke and smoking (Sharif et al., 2019). Many stroke survivors are affected not only by motor disability but also by cognitive impairment (Tatemichi et al., 1994; Pantoni and Salvadori, 2021). Up to 70% of people after a stroke are affected by cognitive deficits, and of those, up to 50% present with attention deficits (Hochstenbach et al., 1998; Loetscher et al., 2019). Attention includes aspects of mental alertness, divided and sustained attention (Sturm et al., 1997). These aspects of attention are mandatory for daily functioning and support recovery after stroke (Robertson et al., 1997; McDowd et al., 2003; Lingo VanGilder et al., 2020). If attention deficits persist for more than 1 year after the stroke, they are considered chronic (Larson et al., 2003) and may affect other higher-order cognitive processes such as memory.

### 1.1 Post-stroke attention deficits, attention networks, and slow cortical potentials

Many brain structures are involved in attention processes, which may be why attention deficits are common among stroke survivors. In the alerting network, the locus coeruleus, frontal cortex, and parietal cortex are mainly involved (Posner et al., 2019). The orienting network involves the superior parietal lobe, the temporal parietal junction, the frontal eye fields, the superior colliculus, and the pulvinar. The executive network comprises the anterior cingulate cortex, the anterior insula, and the basal ganglia (Posner et al., 2019). Deficits in concentration may be caused by reduced cortical connectivity in the alerting and orienting networks (Rosenberg et al., 2016; Posner et al., 2019). Fu et al. (2017) found impaired attention in participants after brainstem stroke (Hoffmann and Watts, 1998). Middle cerebral artery infarction was also associated with attention deficits (Robert Teasell and Hussein, 2016; Rosemann et al., 2017). Likewise, cerebellar infarction (Fan et al., 2019), cerebral parietal infarction (Lunven and Bartolomeo, 2017), basal ganglia hemorrhage (Xiong et al., 2016), and thalamic stroke (Kraft et al., 2015) were reported to lead to attention deficits.

Furthermore, cortical excitability will most likely be affected after a stroke (Huynh et al., 2016). Slow cortical potentials are direct current (DC) shifts measurable at the cortical surface and reflect depolarization or hyperpolarization of cortical cell assemblies, as described in the *threshold regulation of the excitability model* (Elbert, 1993). SCPs were hypothesized to reflect threshold regulation mechanisms of cortical excitability and inhibition (Elbert, 1993). Negative shifts increase the probability of collective neuronal firing, while positive shifts decrease it. They can be event-related to a physical stimulus, a behavioral response, or cognitive and emotional processes (Wyckoff and Strehl, 2011). Therefore, as with other event-related potentials, they might be influenced by the emotional or motivational state (Kleih et al., 2010; Kleih and Kübler, 2013). Their frequency is below 1 Hz, lasting up to several seconds. Amplitudes vary from several to more than 100 microvolts (Birbaumer, 1999). Negative shifts were associated with preparedness, especially when warning stimuli were implemented (S1-S2 paradigms Walter et al., 1964). Attention and short-term memory performance were related to cortical negativity (Elbert, 1993). Several studies found relationships between cortical negativity and behavioral measures such as reaction time or short-term memory performance (Birbaumer et al., 1990; Birbaumer, 1999). For example, a study investigating the effects of SCP neurofeedback training on performance in a signal detection task (button-press task) revealed an inverted U-shaped relation between the SCP amplitude and signal detection performance. Small negative shifts were related to superior performance compared to large negative or positive shifts. Furthermore, participants outperformed control subjects, who did not receive contingent reinforcement for SCP shift regulation. The authors, therefore, suggested that attentional processes are facilitated by negative SCP shifts (Lutzenberger et al., 1979). As the mediothalamic-frontocortical system (Rockstroh et al., 1984) together with motor areas, the posterior parietal cortex, the anterior cingulate cortex, and thalamic nuclei (Hinterberger et al., 2005; Lütcke et al., 2009; Gevensleben et al., 2014) are believed to generate negative SCPs, there seems to be a brain structure overlap between the SCP generation network (Rockstroh et al., 1984) and the alerting and orienting networks (De Bourbon-Teles et al., 2014; Posner et al., 2019).

### 1.2 Treatment of post-stroke attention deficits

Treatment of post-stroke attention deficits commonly includes computerized training (Bogdanova et al., 2016; Amiri et al., 2023). In such training, the participants are instructed to react to stimuli appearing on a computer screen, e.g., for training sustained attention, the picture of a flower presented in a stream of other objects must be detected and indicated by a button press. The theoretical assumption is that by combining stimulus perception with a motor reaction, reorganization of the central nervous system can be activated and supported. In this approach, the brain is activated by processing and reacting to visually perceived stimuli (De Luca et al., 2018). Neurofeedback activates the brain directly and feedback about the cortical activation level is provided (Gevensleben et al., 2011; Schwartz and Andrasik, 2017). Neurofeedback therapy aims to improve a particular state or behavior by bringing a brain activation related to this state or behavior under conscious control. One or more feedback electrodes are placed on a person’s head over a specific area, often at location Cz to assess their brain activity. Changes in brain activation are fed back to the person in a loop, allowing them to learn willful control of this brain activity. The change in brain activity might then lead to an improvement in the behavioral level that is associated with this specific brain activity (Kober et al., 2015; Lavy et al., 2019). Thus, in stroke patients, this neurofeedback loop provides the participant with immediate feedback on cortical modulation and supports voluntary manipulation of the respective cortical network without taking the detour of indirect brain activation through the execution of motor tasks (Larson and Sherlin, 2013). One option to improve attention may be using neurofeedback with slow cortical potentials.

### 1.3 SCP neurofeedback

Numerous studies have demonstrated that SCP shifts can be voluntarily modulated, providing neurofeedback training with long-lasting effects on the related behavior (Rockstroh et al., 1993; Thompson and Thompson, 1998; Kotchoubey et al., 1999; Arns et al., 2009; Mayer et al., 2016). Most studies utilizing SCP neurofeedback therapy have focused on improving symptoms of Attention Deficit Hyperactivity Syndrome (ADHS) by targeting negative SCP shifts to increase cortical excitability. Parents reported significantly fewer problems at home with children diagnosed with ADHS and trained to control their SCPs; teachers confirmed fewer symptoms of hyperactivity, impulsivity, and fewer issues with social behavior (Leins et al., 2007). As measured with respective tests (Zimmermann and Fimm, 2012), attention significantly improved after SCP neurofeedback therapy (Leins et al., 2007). A positive effect of SCP training on hyperactivity and impulsivity was also supported in a sample of more than 70 children diagnosed with ADHS (Gevensleben et al., 2009). For ADHS treatment, SCP neurofeedback training is practical and specific (Sherlin et al., 2010; Arns et al., 2014), even at 6 months follow-up (Gevensleben et al., 2009; Mayer et al., 2016). Barth et al. (2021) conducted a study examining the efficacy of neurofeedback therapy for adults with ADHS. The findings revealed that only 30% of participants could attain SCP control in a 30-session study. The study demonstrated significant improvement in ADHS symptom ratings. These results suggest that adults may find it more difficult than children to learn SCP control and that other non-specific factors may contribute to the observed outcomes.

While there seems to be convincing evidence of SCP neurofeedback to improve attention in ADHS, there is only one study, to our knowledge, that investigated the possible effect of SCP neurofeedback training on the improvement of chronic attention deficits that were caused by stroke (Kleih-Dahms and Botrel, 2023). In that study (Kleih-Dahms and Botrel, 2023), the results seemed encouraging; however, only five patients were included, and participants’ adherence to the neurofeedback training protocol was problematic due to its time-consuming nature. An improvement in quality of life was observed concerning the ability to perform daily living activities. However, data analysis was primarily descriptive, and non-specific effects could not be excluded. Thus, a less time-consuming training protocol with a bigger sample size was planned in this study. We also planned to investigate further the potential impact of improving attention deficits on subjective quality of life.

### 1.4 Psychological consequences of stroke

People after a stroke are often affected by psychological consequences, such as depression (Snaphaan et al., 2009; Robinson and Jorge, 2016) and a deterioration of their subjective quality of life (Robinson-Smith et al., 2000; Donkor, 2018). Due to less capacity for leisure activities, personal relationships might deteriorate (Rochette et al., 2007). Employment for the patient after the stroke, the spouse, or both might change or even end due to the consequences of the stroke (Rochette et al., 2007; Busch et al., 2009), with a specifically higher risk of post-stroke unemployment in patients between 18 and 50 years of age (De Bruijn et al., 2014). Also, cognitive performance affects emotional well-being. People after mild stroke events are often aware of the reduced level of mental capacity and the physical limitations incurred by stroke, which may increase the psychological burden of the event (Lezak, 1988). According to Andersen and colleagues, intellectual impairment after stroke explains 42% of the variance of depressed mood (Andersen et al., 1995). Cumming et al. (2014) showed that cognitive impairment and specifically attention and visuospatial ability were significantly related to perceived quality of life 12 months after the stroke event. Also, Nys et al. (2006) showed cognitive impairment to predict long-term depressive symptoms and quality of life in stroke survivors. The perception that one’s behavior influences the environment (self-efficacy) and being able to handle stroke-induced self-care-related challenges correlated with perceived quality of life and depression (Rochette et al., 2007). To summarize, the quality of life in people with chronic symptoms after a stroke is very likely seriously affected. Learning to regulate SCPs and improving cognitive performance may positively impact emotional state and perceived quality of life (Kleih-Dahms and Botrel, 2023).

Based on the above-summarized research, we hypothesized that people who had a stroke could learn to regulate their SCPs (H1). Secondly, SCP neurofeedback would improve attention as assessed by neuropsychological measures (H2). Thirdly, we predicted a beneficial effect of SCP neurofeedback on emotional state and quality of life (H3). Lastly, we were interested in the potential effect of motivation and emotional state on the ability to regulate the SCPs, as a relation between motivation and SCP control in single subjects was found previously (Kleih-Dahms and Botrel, 2023).

## 2 Methods

### 2.1 Design

We implemented a within-subjects design. Dependent variables were the SCP amplitude for negative and positive SCP shifts, performance in neuropsychological tests, and results of psychological questionnaires, which will be described in detail below. The local Medical Ethics Committee reviewed and approved the study at the University of Würzburg, Würzburg, Germany. The entire methodological procedure was performed in accordance with the Declaration of Helsinki (World Medical Association, 2013).

### 2.2 Participants

Inclusion criteria for this study were a subjective experience of an attention deficit after a stroke and being younger than 85 years. Exclusion criteria were a stroke in the subacute instead of the chronic phase and intake of neuroleptic medication or ongoing adjustment in the medication plan. We recruited *N* = 28 participants through a local newspaper advertisement. Twelve participants dropped out due to time constraints, lack of commitment, fatigue, illness, or another stroke. Six participants dropped out before the second neurofeedback training session, four before the eighth training session, and two before the second neuropsychological assessment. None of the drop-out participants was included in the data analysis. The remaining sample of *n* = 16 included *n* = 3 female and *n* = 13 male participants. The average age was 65.5 (*SD* = 8.25, range = 37–72), and the average time since the stroke event was 4.34 years. Diagnostic details, as described in the medical records provided by the patients, are summarized in Table 1. All participants gave written informed consent before participation.

**Table 1:** Participant number (PN), sex (f = female, m = male), age, lesion side, medical description of a stroke, and time since stroke event (= tsse) in years (y) and months (mo) are displayed for every participant.

PN	Sex	Age	Lesion side	Diagnosis according to the medical report	tsse
6	M	66	Right	Brain stem stroke	1 y 10 mo
8	M	62	Bilateral	Brain stem stroke	2 y 6 mo
10	M	72	Right	Middle cerebral artery infarction	1 y 10 mo
11	M	69	Bilateral	Localized and small medullary infarctions in the Corona Radiata and the semioval center, Lacunar infarction in the thalamic periphery	8 y
12	M	70	Right	Middle cerebral artery infarction	4 y 10 mo
14	M	72	Left	Cerebral parietal ischemia, frontotemporoparietal hemorrhagic infarction	1 y 5 mo
15	F	70	Right	Middle cerebral artery infarction	3 y 7 mo
16	M	72	Left	Middle cerebral artery infarction	1 y 7 mo
18	F	66	Right	Hypertensive hemorrhage in the basal ganglia	8 y 6 mo
19	M	64	bilateral	Middle cerebral artery infarction	2 y 10 mo
21	M	68	Left	Thalamus infarction	1 y 1 mo
22	M	63	Left	Cardial-embolic cerebellar infarction	14 y 4 mo
23	F	37	Right	Transient ischemic attack	1 y 2 mo
25	M	65	Right	Thalamic hemorrhage with ventricular bleed	10 y 2 mo
27	M	66	Left	Middle cerebral artery infarction	10 y 2 mo
28	M	66	Right	Middle cerebral artery infarction with plaque-forming cerebral macroangiopathy	10 mo

Participants identified themselves as female or male as indicated by their biological sex and as European.

### 2.3 Procedure and stimuli

**Figure 1:**
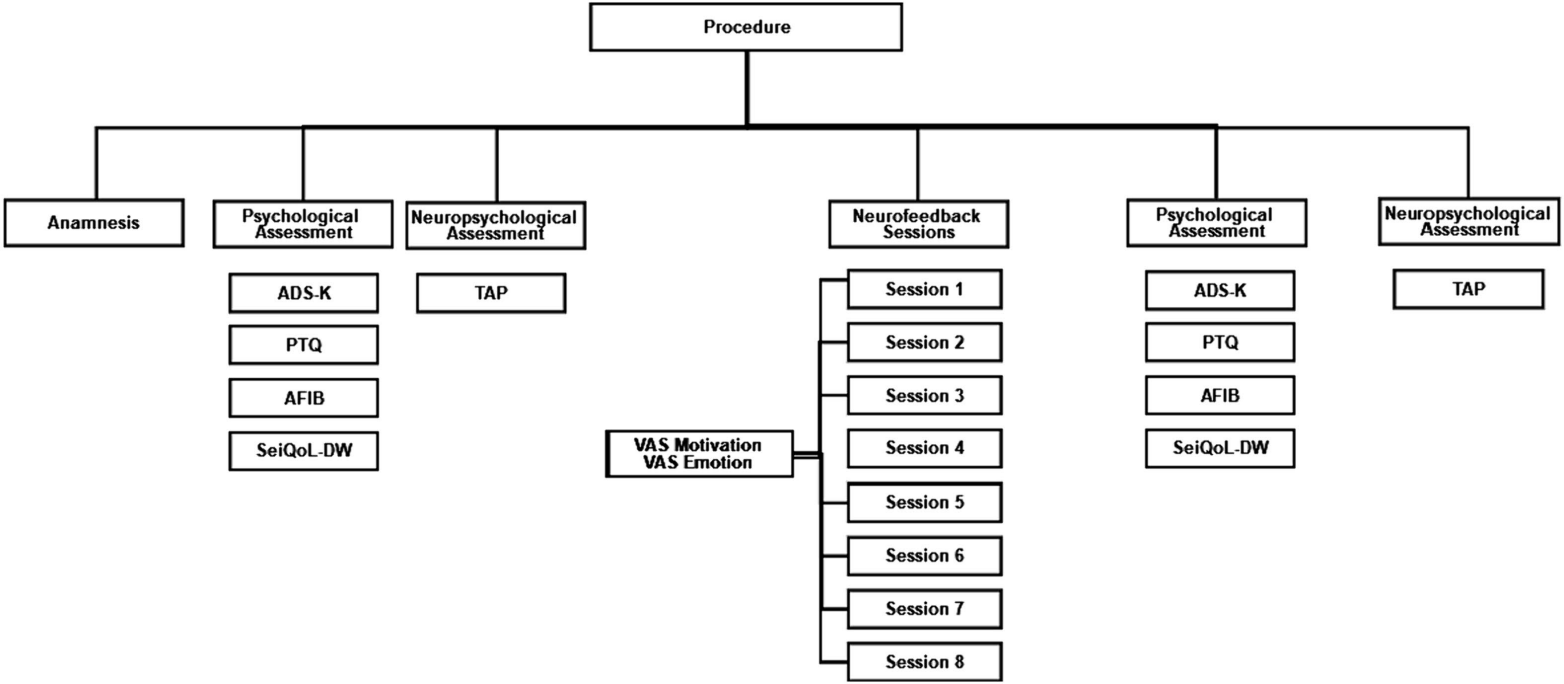
Procedure flow chart. ADS-K, Center for Epidemiological Studies Depression Scale (German: Allgemeine Depressionsskala Kurzform); PTQ, Perseverative Thinking Questionnaire; AFIB, Activities of daily living; SeiQoL-DW, The schedule for the evaluation of individual Quality of Life direct weighting; TAP, Test Battery for Attention Performance.

An anamnestic session was scheduled, and neuropsychological tests and psychological questionnaires were assessed in two sessions before and after SCP neurofeedback (Figure 1). Data acquisition for the psychological tests lasted two and a half hours, including a half-hour break. Participants were asked to complete the questionnaires on their own, or with the help of the experimenter. Participants underwent eight sessions of neurofeedback within three to four weeks. The session duration was approximately 4 hours, including EEG preparation. Each participant completed neurofeedback during a fixed appointment to control for daytime attention fluctuations.

#### 2.3.1 Neuropsychological tests

We assessed attention with the test battery for attention performance (TAP) (Zimmermann and Fimm, 2012). We followed Elbert’s threshold regulation excitability model (Elbert, 1993) to choose relevant dependent variables of the TAP. In his model, cortical negativity is related to attention and short-term memory processes and indicates the resources required for information processing. Furthermore, negative SCP shifts are postulated to correspond to a state of preparedness, especially when warned before a stimulus. Accordingly, we used the TAP phasic alertness parameter (s), the omissions in the TAP working memory test, reaction times and errors in the TAP flexibility subtest, and the omissions in the TAP subtest of divided attention as dependent variables. For the TAP the criteria of validity, objectivity, and reliability were met (Zimmermann and Fimm, 2012). Reliabilities of the subtest were reported for each subtest.

##### 2.3.1.1 The TAP phasic alertness parameter

The *TAP Alertness* test examines alertness under two conditions in four blocks. 1) Intrinsic alertness: A cross appears on the monitor at randomly varying intervals. The subject is instructed to respond quickly by pressing the key. 2) Phasic alertness: The cross is preceded by a cue stimulus (warning tone).

The order of the two conditions within the four blocks is 1–2–2-1. The outcome variable is the phasic alertness parameter. It is calculated as follows (Eq. 1).


(1) $$\mathrm{Phasic}\;\mathrm{alertness}=\frac{\left(\mathrm{RMedia}n_{\mathrm{trials}\;1+4}-\mathrm{RMedia}n_{\mathrm{trials}\;2+3}\right)}{\mathrm{RMedia}n_{\mathrm{trials}\;1+4}}$$

For the TAP alertness test a re-test reliability of 0.81 was reported (Zimmermann and Fimm, 2012).

##### 2.3.1.2 The TAP working memory test

The *TAP Working Memory* test examines how well perception is controlled and the ability to update information in working memory. First, a sequence of numbers is presented. Next, the person is instructed to determine whether the currently given number equals the one given two numbers before (two-back-task, e.g., 3–8-3” press the key”). As omissions in this test indicate a person’s distractibility by irrelevant stimuli, we used omissions as the dependent outcome variable. For the TAP working memory test a re-test reliability of 0.67 was reported (Zimmermann and Fimm, 2012).

##### 2.3.1.3 The TAP flexibility test

In the *TAP Flexibility* (nonverbal, alternating) test, a round and an angular shape are presented randomly on the right or left side of the computer screen. The patient is instructed to alternate between two tasks: 1) Press the key on the side of the round shape and 2) Press the key on the side of the angled shape. As this test measures the ability to focus and shift attention allocation, we were interested in reaction times and errors. For the TAP flexibility test a re-test reliability of 0.83 was reported (Zimmermann and Fimm, 2012).

##### 2.3.1.4 The TAP divided attention test

In the *TAP Divided Attention* (auditory + visual) test, the participant is instructed to simultaneously attend to a visual and an auditory task. In the visual task, a key must be pressed when four randomly moving crosses form a square. In the auditory task, low- and high-pitched tones are presented alternatingly, and the key must be pressed when this sequence is violated. As omissions indicate failure in divided attention, omissions were our dependent variable. For the TAP divided attention test a re-test reliability of 0.44 was reported (Zimmermann and Fimm, 2012).

#### 2.3.2 Psychological questionnaires

*Center for Epidemiological Studies Depression Scale – the Allgemeine Depressionsskala Kurzform (ADS-K).*

To measure possible depressive symptoms, we used the German version of the *Center for Epidemiological Studies Depression Scale – the Allgemeine Depressionsskala Kurzform (ADS-K)* (Hautzinger et al., 2012). The ADS-K is a screening instrument for self-evaluation, which quantifies depressive symptoms. It comprises 15 items to be judged on a 4-point Likert scale. The cut-off value for depressed mood is 17. According to the manual (Hautzinger et al., 2012), psychometric test quality criteria were met. Reliability was reported at 0.81 (Hautzinger et al., 2012).

##### 2.3.2.1 Repetitive thinking – the perseverative thinking questionnaire (PTQ)

The Perseverative Thinking Questionnaire (PTQ) (Ehring et al., 2011) was developed to measure Repetitive Negative Thinking (RNT). The PTQ consists of 15 items covering three aspects of RNT: repetitive thinking, unproductive thinking, and capturing mental capacity. Items were judged on a 4-point Likert scale. Psychometric properties were judged as sufficient and reliability was reported to be 0.95 (Ehring et al., 2011).

##### 2.3.2.2 Activities of daily living – the Aachener functioning item bank (AFIB)

We assessed the activities of daily living (ADL) with the AFIB, an item pool instrument based on the Rasch model (Böcker et al., 2007). Daily functioning covers three different areas: 1) applied cognition, 2) personal care and social activities (inclusion), and 3) physical and movement activities (mobility). We focused on the cognition scale, including 18 items to be answered on a five-point Likert scale. The authors judged the psychometric properties to be sufficient, but test values were not published.

##### 2.3.2.3 Quality of life – the schedule for the evaluation of individual quality of life direct weighting (SEIQoL-DW)

The SEIQoL-DW (Hickey et al., 1996) quantifies the subjective quality of life (QoL) and disease-related response shifts and might indicate successful coping (Carr et al., 2001). First, participants define up to five most important domains for their QoL. Next, they weigh these domains using circular, colored disks that can be rotated around a fixed point, like a pie chart. Satisfaction with each domain is rated on a scale ranging from 0 (not at all satisfied) to 100 (fully satisfied). Finally, the total score is calculated by dividing the indicated satisfaction with each life domain by 100 and then multiplying the result with importance as determined by the size of the pie portion. The result is an index for individual quality of life, which ranges between 0 and 100 (SeiQol-Index). Psychometric data were judged as sufficient, and reliability was reported to be 0.73 (O’Boyle, 1994).

##### 2.3.2.4 Motivation and emotional state – visual analog scales

The visual analog scales (VAS) motivation and emotion range from 0 (not motivated at all, in a bad emotional state) to 10 (very motivated, perfect emotional state) on a 10 cm long horizontal line. Participants indicated their motivation and emotional state before and after each neurofeedback session.

#### 2.3.3 SCP neurofeedback

We used a Lenovo Think Pad Edge E520 with a 15.6-inch screen for data acquisition. An online electroencephalography (EOG) artifact rejection was implemented during neurofeedback training. A neurofeedback trial was rejected if the correlation between the EEG and EOG during training exceeded >0.6. Each abandoned trial was repeated, however, at most three times.

**Figure 2:**
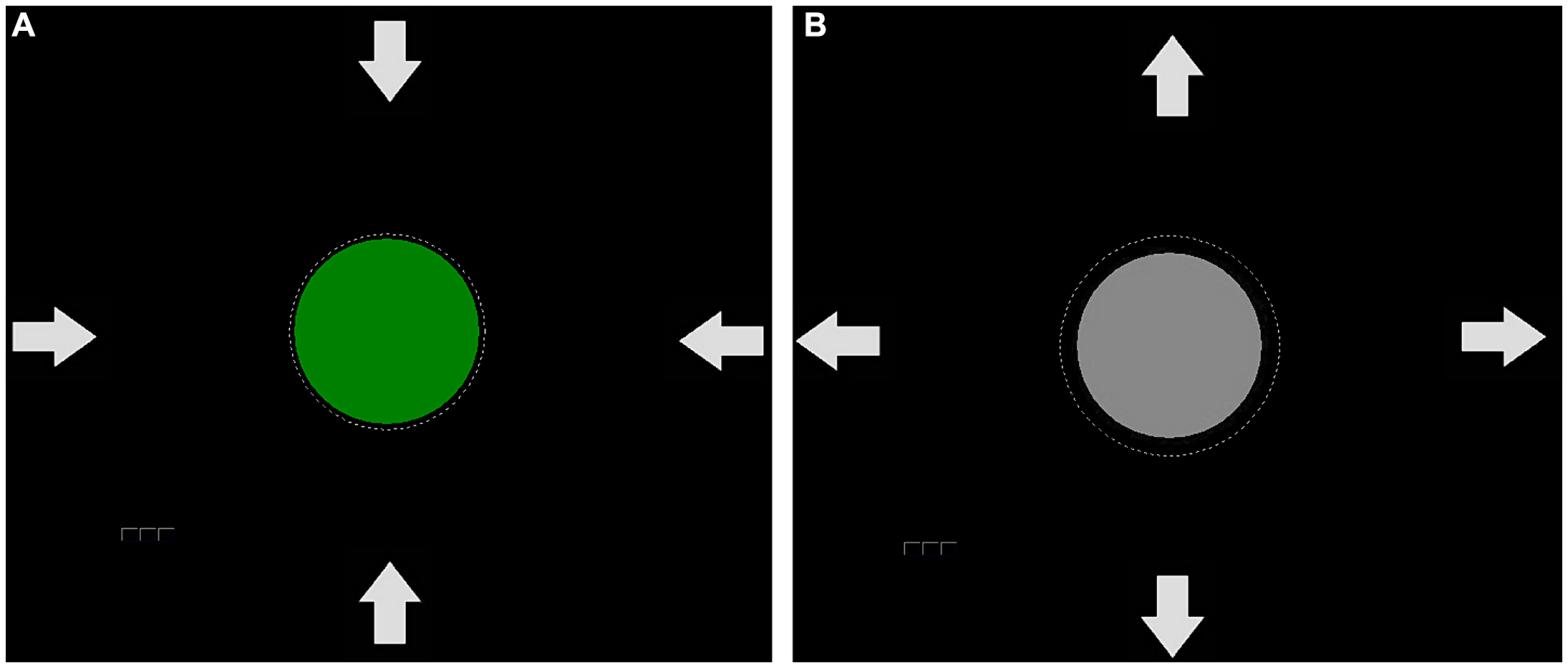
Feedback used in the SCP protocol. The arrows serve as an indication of whether the circle should be enlarged or reduced in size. If a positivity shift was successfully performed, the circle decreased in size and turned green **(A)**. If a negativity trial was necessary, the circle would remain gray **(B)** until the negativity shift occurred and led to an increase in circle size, which would then cause a color change.

To train SCP regulation, participants had to focus on a gray circle in the center of a computer screen (Figure 2). We opted for a circular display for feedback to reduce the chances of eye artifacts. This way, participants did not have to shift their gaze up or down to view the feedback (Kleih-Dahms and Botrel, 2023), which was presented in the center of the screen. The circle’s diameter was 5 cm and changed continuously in size according to the SCP amplitude. The raw EEG signal was used for feedback in the range of −600 μV – 600 μV in the time window of 2000 to 6,000 ms of each trial. In case of activity exceeding this range, the trial was rejected and repeated at the end of the block. In negativity trials, participants were required to increase, and in positivity trials, to decrease the circle size. The required EEG polarity was indicated by a written instruction at the beginning of the trial, and by white arrows of 2 cm in length (Figure 2). The circle turned green when the correct polarity shift was achieved. Negative feedback was not provided. We did not provide instructions on how to change the circle diameter. Instead, we instructed participants to find their strategy. Participants performed eight sessions, each containing 10 blocks. Each block consisted of 50 trials.

To establish a baseline, the average voltage of the 500 ms period before each trial was measured. All brain activation changes were compared to this baseline, and every activity change towards the required polarity was reinforced (circle turned green) without having to reach a certain threshold. After each block, a participant could take a break of individual length. Positive and negative SCP amplitude shift trials were equally balanced in the first five sessions. In the last three sessions, we presented 350 negativity, and 150 positivity trials as negative SCP shifts were hypothesized to be related to attention. After four sessions, participants were instructed to train SCP neurofeedback negativity at home in situations where an increased attentional state would be beneficial. Such cases were, e.g., medical appointments, cooking, and talking to more than one person.

The BioTrace Software package controlled the protocol (MindMedia, The Netherlands). EEG was low-pass (30 Hz), high-pass (0.01 Hz), and notch filtered (50 Hz). EEG was recorded with an Ag/AgCl electrode at Cz and was referenced to the right mastoid at a sampling rate of 256 Hz (NEXUS, MindMedia, The Netherlands). The ground electrode was positioned at C3. Vertical EOG was recorded with bipolar electrodes mounted below and above one eye. Horizontal EOG was recorded with one electrode on the left temple. In addition, one electrode was placed at the left mastoid for online and offline re-referencing to linked mastoids. After electrode preparation, we waited 10 min before starting the SCP neurofeedback training to account for DC drifts. All data were stored as raw files without online artifact correction and exported for data analysis using the Biotrace Software (Mindmedia, The Netherlands).

### 2.4 Data analysis

Data were segmented into 6500 ms long intervals starting with the baseline of 500 ms before trial onset. Each segment was baseline-corrected. Ocular artifacts were corrected using a regression-based approach (Gratton et al., 1983), and the remaining artifacts were excluded via a threshold of ±150 μV. Negativity and positivity trials were averaged separately per individual. Finally, mean SCP amplitudes (μV) for negativity and positivity in the feedback time between 2000 and 6000 ms were exported for statistical analysis using SPSS IMB^®^ version 26.

Data were checked for normal distribution and homogeneity of variances. In case Mauchly’s test for sphericity was significant, we used Huynh-Feldt’s corrected statistics. When conditions for parametric testing were violated, non-parametric alternatives were applied.

### 2.5 Data availability

The datasets analyzed during the current study are available from the corresponding author upon reasonable request.

## 3 Results

### 3.1 SCP regulation

**Figure 3:**
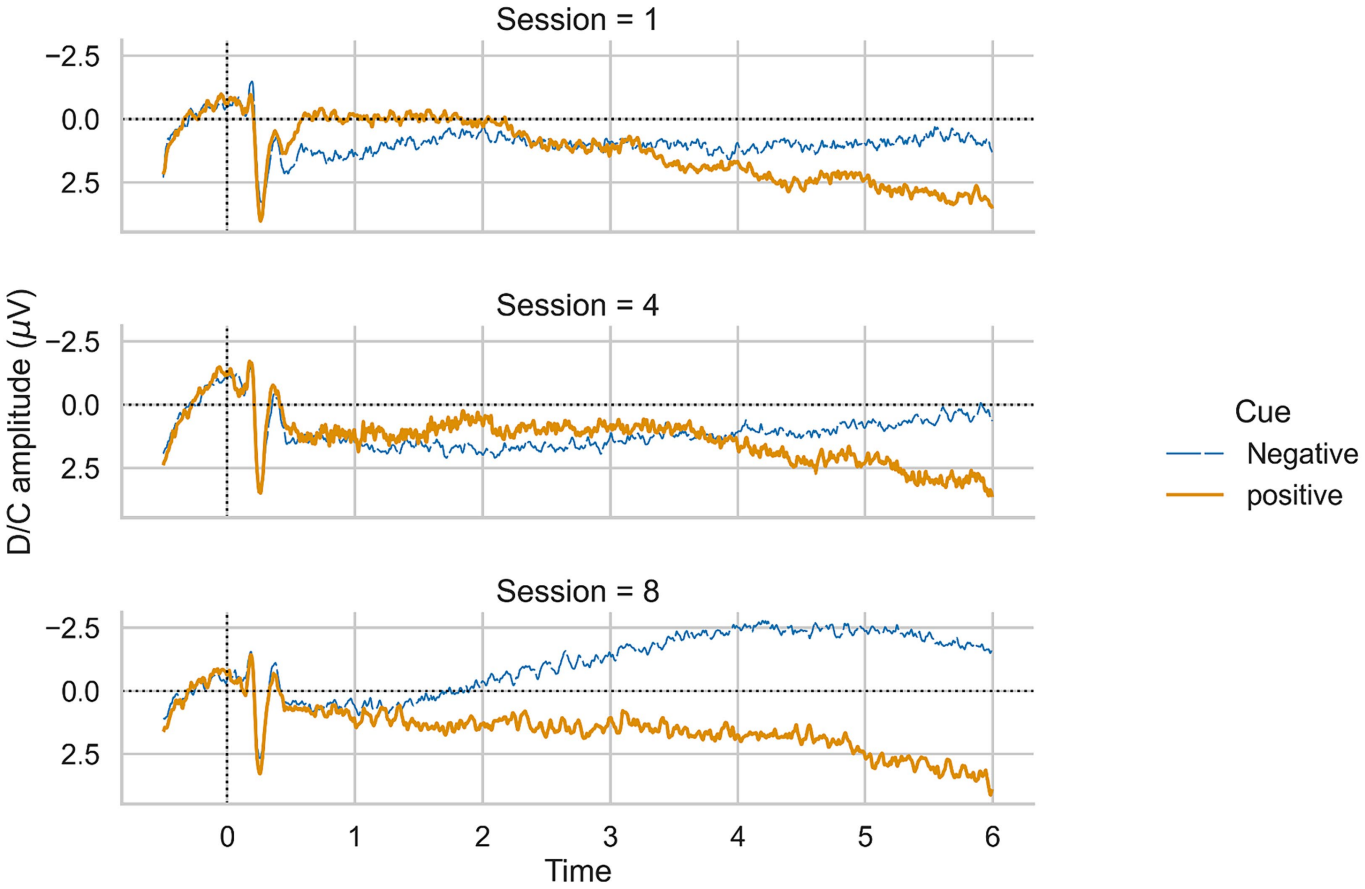
SCP negativity and positivity averaged for all participants and sessions 1, 4, and 8.

To test whether patients learned SCP regulation (H1), we calculated a repeated measures ANOVA with time (sessions one to eight) as the within-subjects factor and the averaged negative SCP amplitude (μV) as the dependent variable. We did not find a significant effect of time on SCP negativity (*F* (7, 105) = 1.88, *p* = 0.08, Figure 3), but observed an increase from session one to eight. Averaged positivity SCP amplitudes did not increase [*F* (7, 105) = 1.61, *p* = 0.14, Figure 3]. Fourteen individuals reported practicing SCP control at home without a neurofeedback device in relevant situations where they wanted to increase their attention. Patients reported mental calculation, mental crossword puzzles, counting, or thoughts about the circle to increase as strategies for SCP negativity. For SCP positivity trials, relaxation, not thinking about anything, observing the circle on the screen, or thinking about the sea were reported. Four participants could not report strategies, neither for SCP positivity nor negativity. One participant reported a change in strategy for negativity trials after the third session, initially thinking about an opening and closing fan, then shifting to an inner observation of objects flying through the air.

To conclude, we could not confirm our first hypothesis. However, we observed a trend toward increased negative SCP amplitudes.

### 3.2 Attention

We found test values below the expected age average (below a T value of 40) in 15 out of 16 participants in at least one TAP subtest. Several participants showed below-average results in multiple subtests (*n* = 6 in four subtests, *n* = 4 in three subtests, and *n* = 5 in one subtest). To test H2 that attention would increase with learning to regulate SCP negativity, we calculated a repeated measures ANOVA for each of the dependent variables with time (pre- versus post-SCP training) as within subjects’ factor and the phasic alertness parameter, omissions in the divided attention test, the reaction time of the flexibility test, and omissions in the working memory test as dependent variables. For evaluating errors in the flexibility test, we used the Wilcoxon test for dependent samples due to violations for parametric testing. For both dependent variables in the flexibility test (reaction time and errors), we only had data of *n* = 15 participants, as Pn 14 did not participate in the second flexibility test due to circulation problems. Results are summarized in Table 2.

**Table 2:** TAP test performances on average and statistical comparison between t1 and t2.

TAP test and test parameter	Analysis	Test statistic	df	Test value	*p*	Effect size *η*^2^
Phasic alertness parameter	RMA	*F*	15	0.31	0.59	0.02
Divided attention omissions	RMA	*F*	15	7.97	0.01*	0.35
Flexibility RT	RMA	*F*	14	8.31	0.01*	0.37
Flexibility errors	Wilcoxon	*Z*	14	1.88	0.06	0.24
Working memory omissions	RMA	*F*	15	2.17	0.16	0.13

RMA, repeated measures ANOVA, * significant *α* = 0.05.

In the TAP divided attention test, participants omitted, on average, 4.38 responses (*SD* = 2.87) at t1 and significantly less at t2 (*M* = 3.37, *SD* = 2.65, Table 2). In the TAP flexibility test, participants needed 1586.65 ms to respond (*SD* = 843.89) at t1 and reacted significantly faster at t2 (*M* = 1237.07 ms, *SD* = 583.12). Error rates improved from 6.81 (*SD* = 6.12) at t1 to 4.33 errors (*SD* = 5.47) at t2. However, this improvement was not significant. Thus, our second hypothesis of improved attention after SCP training was confirmed for divided attention and flexibility.

### 3.3 Emotional state and quality of life

To test H3 that SCP neurofeedback positively influences emotional state and quality of life, we used repeated measures ANOVAs with time of measurement as within subjects’ factor and the ADS-K, PTQ, SeiQoL, and AFIB data as dependent variables. All results are depicted in Table 3. ADS-K scores improved between t1 and t2. However, despite this tendency for SCP training to have a positive influence on the emotional state, four patients reported ADS-K values above the cut-off of 17 at t2.

**Table 3:** Mean raw values (M) and standard deviations (SD) Center for Epidemiological Studies Depression Scale – the Allgemeine Depressionsskala Kurzform (ADS-K), the Perseverative Thinking Questionnaire (PTQ), the Schedule for the Evaluation of Individual Quality of Life direct weighting (SeiQol) and the Aachener Funktionsitembank (AFIB) before (t1) and after (t2) SCP neurofeedback training.

test	t1 M (SD)	t2 M (SD)	*F* (1,15)	*p*
ADS-K	17.06 (7.38)	13.81 (7.07)	4.05	0.06
PTQ	24.06 (11.61)	23.31 (12.11)	0.24	0.63
SeiQoL	61.44 (16.43)	63.96 (13.70)	0.63	0.44
AFIB	44.81 (9.50)	46.94 (6.81)	1.57	0.23

Statistical test value = F, significance.

Neither Repetitive Thinking (PTQ) nor the subjective quality of life (SeiQoL) or daily life functioning (AFIB) changed between t1 and t2. H3 must be rejected as we found no significant effect of SCP neurofeedback on psychological variables.

Participants were highly motivated (*M_session1_* = 7.93, *SD* = 1.94; *M_session8_* = 7.33, *SD* = 2.55; *F* (7,105) = 0.73, *p* = 0.65, η^2^ = 0.05) and in good emotional state (*M_session1_* = 7.33, *SD* = 2.11; *M_session8_* = 7.21, *SD* = 2.63; *F* (7,105) = 0.93, *p* = 0.49, η^2^ = 0.06) throughout the training. To test whether motivation and emotional state were related to the ability to regulate the SCP amplitude voluntarily, we correlated VAS motivation and VAS emotion scores assessed before training with the averaged SCP amplitude during negativity trials in all sessions. Spearman rho correlations between motivation and SCP negativity ranged between 0.36 (*p* = 0.17, session 1) and − 0.16 (*p* = 0.54, session 5). The relation between emotional state and SCP negativity ranged between 0.43 (*p* = 0.10, session 1) and − 0.12 (*p* = 0.66, session 5). None of the correlations were found to be significant, indicating no relationship between motivation or emotional state and SCP negativity.

### 3.4 Post-hoc analysis with responders and non-responders

We post-hoc compared responders to non-responders concerning the learning of SCP regulation toward negativity. We selected responders and non-responders by plotting the learning curves of each participant based on the average activation value for the SCP negativity trials per session. We then added the linear prognosis function following the equation a+bx given that:


$$a=\bar{y}-b\bar{x}$$

And


$$b=\frac{\Sigma \left(x-\pi \right)\left(y-\bar{y}\right)}{\Sigma {\left(x-\bar{x}\right)}^{2}}$$

We calculated the according *R*^2^ value for each linear prognosis function. We classified the participant as a responder if the linear prognosis function pointed toward negativity changes in SCP activation. If the linear prognosis function indicated positive changes in SCP activation or flat lines close to zero, we classified participants as non-responders. In the responder group, we chose the participants with the highest R^2^ values of at least 0.10 such that the values could not be rounded to zero. We identified n = 5 responders (*n* = Pn 06, Pn 14, Pn 19, Pn 22, and Pn 23). To allow for a comparison of equal numbers, those *n* = 5 non-responders were selected who presented with the lowest *R*^2^ values (Pn 10, Pn 11, Pn 12, Pn 16, and Pn 21).

#### 3.4.1 SCP regulation in responders and non-responders

**Figure 4:**
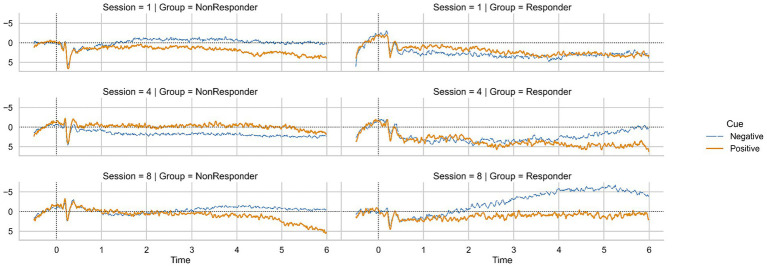
SCP positivity and negativity in sessions 1, 4, and 8 for non-responders and responders.

Due to the small sample size of responders and non-responders, we analyzed all following comparisons solely with non-parametric statistics. The average SCP regulation for negativity and positivity in responders and non-responders for sessions one, four, and eight are depicted in Figure 4.

To investigate whether the SCP regulation improvements that led to the classification as a responder were significant, we calculated a Friedman ANOVA. We found a significant increase of SCP negativity over sessions one to eight in responders (Friedman ANOVA, *F* (7) = 20.53, *p* = 0.01, Figure 4). Average negativity activation changed from *M* = 2.82 μV (*SD* = 3.60) in session one to *M* = −1.99 μV (*SD* = 3.79) in session eight. Concerning SCP positivity, we found no significant changes in responders (Friedman ANOVA, *F* (7) = 11.40, *p* = 0.12, Figure 4). In non-responders, we found no difference in SCP negativity (Friedman ANOVA, *F* (7) = 5.30, *p* = 0.62, Figure 4) or positivity (Friedman ANOVA, *F* (7) = 3.80, *p* = 0.80, Figure 4) from session one to session eight.

#### 3.4.2 Attention in responders and non-responders

We compared the TAP test parameters at t1 and t2 within each group using the Wilcoxon test and between groups at t2 using the Mann–Whitney U test. In responders, we found a decrease between t1 and t2 concerning omissions in the TAP divided attention test (Z (4) = −2.02, *p* < 0.05). All other comparisons did not reveal significant changes in behavioral measures in responders or non-responders within groups (t1 vs. t2) or between groups at t2 (see Table 4).

**Table 4:** TAP test performance statistical comparison within the groups of responders and non-responders at t1 and t2 and between responders and non-responders at t2.

TAP test and test parameter	Analysis	Test statistic	Test value	*p*	Comparison	Group
Phasic alertness parameter	Wilcoxon	*Z*	0.41	0.68	t1 t2	R – R
Divided attention omissions	Wilcoxon	*Z*	2.02	0.04	t1 t2	R – R
Flexibility RT	Wilcoxon	*Z*	1.46	0.14	t1 t2	R – R
Flexibility errors	Wilcoxon	*Z*	1.46	0.14	t1 t2	R – R
Working memory omissions	Wilcoxon	*Z*	0.00	1.00	t1 t2	R – R
Phasic alertness parameter	Wilcoxon	*Z*	0.00	1.00	t1 t2	NR – NR
Divided attention omissions	Wilcoxon	*Z*	1.60	0.11	t1 t2	NR – NR
Flexibility RT	Wilcoxon	*Z*	1.84	0.07	t1 t2	NR – NR
Flexibility errors	Wilcoxon	*Z*	1.48	0.14	t1 t2	NR – NR
Working memory omissions	Wilcoxon	*Z*	1.46	0.14	t1 t2	NR – NR
Phasic alertness parameter	Mann–Whitney U	*Z*	0.74	0.55	t2	R – NR
Divided attention omissions	Mann–Whitney U	*Z*	−0.11	0.92	t2	R – NR
Flexibility RT	Mann–Whitney U	*Z*	−0.62	0.56	t2	R – NR
Flexibility errors	Mann–Whitney U	*Z*	0.50	0.73	t2	R – NR
Working memory omissions	Mann–Whitney U	*Z*	−0.94	0.42	t2	R – NR

R, responder; NR, non-responder.

#### 3.4.3 Emotional state and quality of life in responders and non-responders

Wilcoxon test for the within-group comparison at t1 and t2 and Mann–Whitney U test for the between-group comparison at t2 revealed no statistical differences within or between groups (see Table 5).

**Table 5:** Statistical comparison within the groups of responders and non-responders at t1 and t2 and between responders and non-responders at t2 for the Center for Epidemiological Studies Depression Scale – the Allgemeine Depressionsskala Kurzform (ADS-K), the Perseverative Thinking Questionnaire (PTQ), the Schedule for the Evaluation of Individual Quality of Life direct weighting (SeiQol) and the Aachener Funktionsitembank (AFIB) before (t1) and after (t2) SCP neurofeedback training.

test	Analysis	Test statistic	Test value	*p*	Comparison	Group
ADS-K	Wilcoxon	Z	−0.67	0.50	t1 t2	R – R
PTQ	Wilcoxon	Z	−0.73	0.47	t1 t2	R – R
SeiQoL	Wilcoxon	Z	−0.27	0.79	t1 t2	R – R
AFIB	Wilcoxon	Z	1.21	0.23	t1 t2	R – R
ADS-K	Wilcoxon	Z	−1.83	0.07	t1 t2	NR – NR
PTQ	Wilcoxon	Z	−1.08	0.28	t1 t2	NR – NR
SeiQoL	Wilcoxon	Z	1.83	0.07	t1 t2	NR – NR
AFIB	Wilcoxon	Z	0.00	1.00	t1 t2	NR – NR
ADS-K	Mann–Whitney U	Z	1.16	0.31	t2	R – NR
PTQ	Mann–Whitney U	Z	1.79	0.10	t2	R – NR
SeiQoL	Mann–Whitney U	Z	−0.53	0.69	t2	R – NR
AFIB	Mann–Whitney U	Z	0.60	0.69	t2	R – NR

R, responder; NR, non-responder.

Motivation and emotional state were not related to the ability of SCP regulation neither in responders nor non-responders, as calculated by Kendall’s Tau correlation coefficient. Motivation and SCP negativity were correlated with values ranging between *Τ* = 0.60 (*p* = 0.14, session 1) and *Τ* = −0.74 (*p* = 0.08, session 8) in responders and with values ranging between *Τ* = 0.32 (*p* = 0.45, session 1) and *Τ* = −0.60 (*p* = 0.14, session 2) in non-responders. The relation between emotional state and SCP negativity ranged between *Τ* = 0.80 (*p* = 0.05, session 4) and *Τ* = −0.80 (*p* = 0.05, session 8) in responders and *Τ* = 0.20 (*p* = 0.62, session 1) and *Τ* = −0.80 (*p* = 0.05, session 2) in non-responders.

## 4 Discussion

We investigated the possibility of training people with chronic stroke-related attention deficits to voluntarily control their slow cortical potentials through a neurofeedback-based intervention (Sherlin et al., 2011). Within eight sessions, we found a non-significant increase in the amplitude of SCPs with negative polarity. On the behavioral level, performance in two TAP attention tests (Zimmermann and Fimm, 2012) improved compared to before SCP neurofeedback training. Upon comparison between responders and non-responders, improved divided attention was observed in the former group, whereas the TAP flexibility test yielded non-significant results. According to Elbert’s *Threshold Regulation of Excitability Model* (Elbert, 1993), a behavioral change in attention would be preceded by a difference in the DC range of the EEG frequency spectrum, i.e., a frequency repeatedly linked to attention. In this study, the divided attention test seemed most sensitive to improvements in attention after the SCP neurofeedback training. Increased neuronal excitability supports the ability to react to several simultaneously presented stimuli. However, we must mention the possibility that unspecific effects, such as participation in the study, irrespective of the SCP training, might have led to the behaviorally measured improvements in attention presented here (Barth et al., 2021). Unspecific effects could be the reason behind finding fewer significant behavioral tests in the responders’ group as compared to using the entire sample for data analysis.

This study strengthens our previous finding that patients with chronic attention deficits after stroke can learn to control their SCPs (Kleih-Dahms and Botrel, 2023). Interestingly, similar strategies were reported to control SCPs by using relaxing thoughts to control SCPs toward positivity and more action-oriented thoughts to control SCP negativity. It must be emphasized that our sample was diagnosed with chronic stroke-related deficits that lasted from one to several years. In chronic stroke patients, by definition, cognitive improvement is questionable. None of our participants received other therapies to improve attention during our intervention, nor have they received therapeutic interventions that might have led to the amelioration of attention deficits. However, Loetscher et al. (2019) conducted a review that showed that interventions for cognitive enhancement following a stroke were only effective in the short term and resulted in improvements in divided attention immediately after treatment. Therefore, it is possible that our results concerning improvements in divided attention may not have a long-lasting effect. Contradictory to previous work (Mayer et al., 2013; Kleih-Dahms and Botrel, 2023), we did not find improvements in quality of life or the ability to handle daily life situations. Some participants reported more worries and a lower level of perceived quality of life after the neurofeedback therapy compared to before. The neurofeedback intervention may have activated negative thoughts about the stroke event, its consequences, and worries about the future (Upatel and Gerlach, 2008). Then again, some participants were proud to contribute to research, which might have strengthened perceived self-efficacy (Bandura, 1977) and positively affected depressive symptoms and daily functioning (Robinson-Smith et al., 2000). However, there are no significant findings that suggest the feelings of contributing to research have any impact on the subjective quality of life reported by the participants. One obvious difference between this study and our previous work (Kleih-Dahms and Botrel, 2023) is the number of neurofeedback sessions; it is possible that a more intense training schedule with more than eight sessions (20 in the previous work) would have affected perceived quality of life.

### 4.1 Limitations

Out of the 28 patients who were recruited, 12 of them dropped out due to personal or health-related reasons. We faced technical challenges with the neurofeedback software (Biotrace by Mind Media, the Netherlands). The software was unreliable, which caused several session restarts. Notably, restarts of sessions occurred only in those participants who dropped out due to time constraints, and we, therefore, cautiously conclude that we lost participants due to a sub-optimal neurofeedback software. According to the company, the instability issues were resolved in subsequent software versions.

Some participants did not adhere to the protocol because of the subjective burden imposed by the number of sessions. This challenges a therapeutic procedure that requires longer-term participation or shorter sessions. The training comprised eight neurofeedback sessions. Albeit the optimal number of training sessions has yet to be defined, some studies recommend 20 sessions or more (Sherlin et al., 2011; Kleih-Dahms and Botrel, 2023). Shorter sessions with fewer trials over a more extended period may increase intra-session adherence to the protocol but bear the risk of dropout due to the then extended total number of sessions (Kleih-Dahms and Botrel, 2023). The validity of our results is limited due to the small remaining sample we had. For a study like ours, assuming a small to medium effect, a power of 0.80, α = 0.05, and one group assessed pre and post SCP neurofeedback, according to G*Power Software (Faul et al., 2007), 34 participants would have been required. We are far from this ideal number of participants.

We also must consider possible training effects that might have influenced the behavioral data presented here. According to the TAP test manual (Zimmermann and Fimm, 2012), there may be a training effect in TAP tests that could contribute to a 7 % increase in performance. However, with 4 weeks or more between measurements, such training effects are unlikely (Zimmermann and Fimm, 2012).

### 4.2 Rehabilitation options for people with chronic attention deficits after stroke and the potential of neurofeedback therapy

One might argue that using neurofeedback is quite a time-consuming and complex intervention. Neuropsychological post-stroke interventions for cognitive rehabilitation already exist (Champod et al., 2020) and render other, more complex interventions superfluous. Amongst these is computerized training, for which, in some studies, beneficial effects were found (De Luca et al., 2018); however, a recent meta-analysis found no superiority of computer-based interventions compared to treatment as usual in cognitive rehabilitation after stroke (Mingming et al., 2022). Treatment as usual often includes a neuropsychological assessment of cognitive deficits and a rehabilitation schedule based on the identified deficits (Toniolo, 2011). Attention Performance Training (APT) is a hierarchical treatment program (Sohlberg and Mateer, 1987) shown to improve attention in post-stroke participants (Barker-Collo et al., 2009). Newer interventions suggest using virtual reality applications to enhance possible rehabilitation effects by fostering immersion (Larson et al., 2014). All these treatments require no willful change of brain activation, such as neurofeedback interventions. Therefore, neurofeedback has the potential to directly impact activation and support neuronal plasticity. However, neurofeedback is not integrated into the healthcare system for post-stroke cognitive rehabilitation due to insufficient evidence of its impact. Studies are relatively scarce with small sample sizes and often lack control groups (Renton et al., 2017). In their meta-analysis, Renton and colleagues (Renton et al., 2017) concluded that neurofeedback offers a beneficial intervention method. More research is required to clarify the dose–response relationship and address long-term effects using follow-up measurements. Since then, newer neurofeedback research found improved connectivity of brain networks in chronic stroke patients (Giulia et al., 2021).

## 5 Conclusion

Individuals experiencing ongoing attention deficits after a stroke may benefit from SCP neurofeedback therapy, but adhering to a treatment plan that involves multiple sessions can be challenging. Those who respond positively to the therapy may witness improvements in divided attention; nevertheless, it remains unclear whether these improvements are long-lasting. In order to draw accurate conclusions regarding the benefits of SCP neurofeedback for the treatment of chronic post-stroke attention deficits, it is crucial to replicate the results in a larger group of participants and carry out studies that meet higher methodological standards. At the very least, a control group should be included to control for potential unspecific effects.

## Data availability statement

The raw data supporting the conclusions of this article will be made available by the authors, without undue reservation.

## Ethics statement

The studies involving humans were approved by Medical Ethics Committee of the University of Würzburg. The studies were conducted in accordance with the local legislation and institutional requirements. Participants gave written consent to this study.

## Author contributions

SK: Conceptualization, Formal analysis, Investigation, Methodology, Project administration, Supervision, Writing – original draft. LB: Data curation, Investigation, Software, Visualization, Writing – review & editing.
